# Expressive Flexibility and Dispositional Optimism Contribute to the Elderly’s Resilience and Health-Related Quality of Life during the COVID-19 Pandemic

**DOI:** 10.3390/ijerph18041698

**Published:** 2021-02-10

**Authors:** Alberto Sardella, Vittorio Lenzo, George A. Bonanno, Giorgio Basile, Maria C. Quattropani

**Affiliations:** 1 Department of Clinical and Experimental Medicine, University of Messina, 98125 Messina, Italy; maria.quattropani@unime.it; 2 Department of Social and Educational Sciences of the Mediterranean Area, “Dante Alighieri” University for Foreigners of Reggio Calabria, 89125 Reggio Calabria, Italy; v.lenzo@unidarc.it; 3 Department of Clinical Psychology, Teachers College, Columbia University, New York, NY 10032, USA; bongeoa@gmail.com; 4 Department of Clinical and Experimental Medicine, School and Unit of Geriatrics, University of Messina, 98125 Messina, Italy; giorgio.basile@unime.it

**Keywords:** quality of life, physical component summary, mental component summary, clinical psychology, dispositional optimism, expressive flexibility, elderly, COVID-19, public health

## Abstract

The COVID-19 outbreak had a negative impact on psychological status among elderly subjects, negatively affecting their health-related quality of life (HRQoL). Psychological factors that promote resilience might beneficially contribute also to promoting a better HRQoL among elderly subjects. The main purpose of the present study was to investigate the contribution of dispositional optimism and expressive flexibility on the HRQoL of elderly outpatients during the COVID-19 outbreak. The outpatients were recruited from October 2018 to October 2019, and then followed-up during April 2020, by evaluating their HRQoL. The baseline sample consisted of 141 elderly outpatients (mean age 80.31 ± 6.84 years); the final number of outpatients included in the follow-up evaluation was 104 (mean age 80.26 ± 6.39). Univariate and multivariate linear regressions were developed to explore significant associations with the physical and mental component of HRQoL. Baseline dispositional optimism was a predictor of the mental component of HRQoL at follow-up; the flexible suppression of emotional expression was a predictor of the physical component of HRQoL at follow-up. From a psychogeriatric perspective, the accurate assessment of psychological factors, such as dispositional optimism and expressive flexibility, might help physicians and psychologists to recognize additional patients’ vulnerabilities during the current emergency.

## 1. Introduction

Health-related quality of life (HRQoL) is a multidimensional construct, which specifically embraces health-related aspects of individual well-being. Physical and mental components are acknowledged as indexes of HRQoL, as well as the subjective perception of their effect on daily life [1]. The investigation of HRQoL has progressively gained interest in the context of elderly subjects, since the quality of life among older people tends to decline as they age, due to the increased risk of developing chronic medical conditions [2,3]. Besides the occurrence or the worsening of multimorbidity status [4], the reduction of elderly’s HRQoL is additionally exacerbated by the presence of psychological distress, as supported by several studies involving older adults [5,6,7].

COVID-19 is an infectious disease caused by a novel coronavirus, namely Severe Acute Respiratory Syndrome Coronavirus-2 (SARS-COV-2). The principal symptomatology ranges from mild symptoms such as dry cough, fever, headache, myalgia, or fatigue to more severe symptoms such as dyspnea or pneumonia. To compensate for the lack of vaccines, alongside progressively more consolidated pharmacological therapies, several strict non-pharmacological strategies have been implemented to face the pandemic, such as isolation, social distancing, and quarantine, which have proved necessary but, at the same time, have been a source of social and psychological burden [8].

In line with an aging-oriented perspective, elderly subjects are denoted as one of the most vulnerable and at risk categories during the COVID-19 pandemic [9]. The progressive lockdown imposed by national governments represented a challenging condition for elderly subjects, who not only had to face the worry and the fear of contagion, but also had to cope with the distressing experience of quarantine and isolation from relatives and friends [10,11].

Recent studies have highlighted that the COVID-19 emergency had a negative impact on psychological status among elderly subjects, negatively affecting their HRQoL [12,13]. The implementation of strategies to improve the HRQoL of the elderly subjects has progressively represented a crucial goal in recent months. At the same time, the identification of psychological protective features has gained increasing interest. In line with this perspective, factors that promote individual resilience might beneficially contribute also to promoting also a better HRQoL, including among the elderly population [14]. In fact, resilience is usually defined as positive individual effort to overcome difficulties [15], and it appears particularly significant among the elderly, who often have to face several age-related physical, psychological and social issues, with a negative impact on their HRQoL [16].

Dispositional optimism is a widely investigated psychological construct, which has been recognized as able to promote resilience, as well as a positive aging adaptation. According to the original theoretical perspective of optimism advanced by Scheier and Carved [17], each person diversifies their behaviors based on the positive or negative expectations they have. In the presence of positive expectations, the behavior will be characterized by effort and commitment to reach the goal; contrariwise, in the presence of negative expectations, the behavior will be characterized by less effort and commitment. Therefore, optimistic people will be more likely to implement more coping strategies to overcome problems; conversely, pessimistic people exhibit a progressive disengagement in overcoming difficulties, precisely because they consider them insurmountable.

The investigation of dispositional optimism has raised increasing interest in the setting of several medical conditions. Indeed, subjects with higher levels of dispositional optimism have been previously described as more inclined to adopt healthy behaviours (e.g., avoiding smoke or performing physical activity), in order to ameliorate their cardiovascular health [18], and improve their quality of life [19]. Furthermore, the beneficial effects of an optimistic life orientation have been highlighted in patients suffering from chronic neurological [20,21], and metabolic diseases [22].

Emotion regulation is considered a further psychological factor able to promote individual resilience and psychological adjustment, which can positively influence the subject’s HRQoL [23]. Coping and emotion regulation should not be considered merely as beneficial or maladaptive; in this context, the importance of flexibility in emotion regulation (namely, expressive flexibility) has been suggested as a relevant regulatory factor, involving enhancement and suppression abilities [24]. In line with this theoretical framework, individual adaption and mental health depend on one’s ability to flexibly enhance or suppress emotions under situational demands. Accordingly, enhancement refers to the increased emotional reactivity to contextual situations; conversely, suppression describes the relative reduction of own emotional expression in specific contexts [25]. Previous research has highlighted that a better expressive flexibility is associated with lower levels of psychopathological symptoms and healthier psychological adjustment among a community population [26,27,28] and healthcare professionals [29].

The COVID-19 pandemic has severely affected the HRQoL of people worldwide, including Italy [30,31]; in this emergency, the elderly were denoted as one of the most vulnerable populations. In line with these theoretical conceptualizations, we might hypothesize that dispositional optimism and expressive flexibility might differently contribute to the HRQoL of elderly subjects, jointly with common age-related clinical factors such as cognitive status and frailty. The association between dispositional optimism and HRQoL is still poorly investigated among the elderly population; additionally, the evaluation of dispositional optimism has been often circumscribed to outcomes related to specific diseases. Similarly, the majority of the studies aimed at evaluating expressive flexibility involved non-clinical populations. Consistently, to the best of our knowledge, the investigation of expressive flexibility in the elderly population is an original topic of research, as is its association with HRQoL in the elderly population.

In the light of these introductory premises, the principal purpose of our study was to explore the contribution of dispositional optimism and expressive flexibility on the HRQoL of elderly subjects during the COVID-19 outbreak.

## 2. Materials and Methods

### 2.1. Study Design and Setting

The study collects both cross-sectional and longitudinal evidence, as described in the “Participants and Procedures” section below. The Geriatric Outpatients Clinic of the University Hospital in Messina (Italy) was the clinical setting of the study.

### 2.2. Participants and Procedure

Subjects age ≥65 were assessed for inclusion; we chose to consider this range of age since it represents the common age of access to geriatric clinics. The recruitment was carried out during the outpatients’ scheduled visits.

The procedure was based on a cognitive and physical assessment, and an additional psychological interview. We wanted the entire assessment to be understandable and feasible for the outpatients; therefore, we included outpatients who did not show a severe neurocognitive disorder, according to the Diagnostic and Statistical Manual of Mental Disorders (DSM-5) diagnostic criteria [32]; we additionally included outpatients who did not show severe functional and/or sensory deficits. Consistently, those outpatients exhibiting a Mini Mental State Examination (MMSE) score ≤12 were excluded; similarly, those outpatients presenting on wheelchairs, those who were not able to walk, and those showing severe limitations in the upper limbs were excluded. Ultimately, those outpatients who reported severe visual and/or hearing impairments were excluded. The choice to exclude outpatients with the above-mentioned physical deficits was additionally justified because the evaluation of frailty status consisted of the assessment of physical performances, besides other variables, as explicated in depth below.

The procedure was agreed with an experienced geriatrician and an experienced clinical psychologist.

Baseline cognitive, physical, functional, and psychological scores were collected from October 2018 to October 2019.

HRQoL was also longitudinally explored in April 2020, during the first national lockdown caused by the COVID-19 outbreak. Precisely, due to the progressive worsening of the COVID-19 emergency, and due to the consequent suspension of the Geriatric Outpatient Clinic activity, the evaluation of the HRQoL was carried out by telephone interviews. In order to avoid potential rater-related bias, a single trained psychologist conducted the phone calls and administered the related scales. The phone calls were made during the morning hours, with an average of five phone calls per day.

### 2.3. Ethics Statement

Each procedure completed in this study was in accordance with the ethical standards of our institutional research committee, and with the 1964 Helsinki Declaration and its later amendments. Informed consent was collected for all the participants. The Ethics Committee of the University Hospital of Messina approved the protocol of this study (Prot. 23/19).

### 2.4. Measures and Instruments

We employed the MMSE for the screening of cognitive status [33]. We adjusted the raw scores for subjects’ age and education, using available normative data for the Italian population [34].

Physical performances were measured in terms of 4-m gait speed (expressed in meter per second) and handgrip strength (expressed in kilograms, measured by a Jamar dynamometer).

We assessed frailty status by calculating a 35-deficit Frailty Index (FI), in line with the standard procedure [35]. The FI expresses the ratio of deficits present to the total number of deficits considered; the higher the number of deficits detected, the more severe the frailty grade. According to this deficit accumulation model, subjects with a FI ≥ 0.25 are commonly classified as frail [36]. A summary of the variables that were checked for the calculation of the FI is provided as Supplementary Material (Table S1).

The Short Form-12 (SF-12) questionnaire was administered to evaluate HRQoL [37]. SF-12 is a 12-item questionnaire assessing different quality of life related aspects (e.g., physical functioning, limitations due to physical health, pain, vitality, social functioning, and limitations due to emotional problems). The questionnaire provides two synthetic indexes, the Physical Component Summary (PCS) and the Mental Component Summary (MCS), which are related to individual physical state and mental state, respectively. Higher scores correspond to higher levels of HRQoL.

Dispositional optimism was assessed through the revised version of the Life Orientation Test (LOT-R) [38]. The LOT-R is a 10-item questionnaire based on a 5-point Likert scale ranging from 1 (“strongly disagree”) to 5 (“strongly agree”). Three items are phrased positively, thus oriented to optimism (1, 4 and 10), and three are phrased negatively, referring to an inverse direction of optimism (3, 7 and 9). The remaining four items (2, 5, 6 and 8) are distractors, and they are not scored. Higher scores reflect a greater expectation of positive results. We administered the Italian version of the LOT-R, which reaches a Cronbach alpha of 0.81 [39].

Expressive flexibility was evaluated through the Flexible Regulation of Emotional Expression (FREE) scale, which is a 16-item self-report questionnaire assessing the ability to enhance and suppress emotions across different hypothetical contexts [24]. Each item corresponds to a hypothetical scenario, regarding positive or negative emotions. The subject rates the items on a 6-point Likert scale ranging from 1 (“unable”) to 6 (“very able”), based on how the subject would be able to “be even more expressive than usual of how you were feeling”, and how the subject would be able to “conceal how you were feeling”. The FREE scale provides two indexes, namely the ability to enhance emotional expression (FREE Enhancement) and the ability to suppress emotional expression (FREE Suppression), which are expressed by two sub-scores. An overall score is additionally provided, which is a global measure of expressive flexibility (FREE Total). Higher scores correspond to better expressive flexibility.

### 2.5. Data Analysis

Data were analyzed using IBM SPSS 22 (IBM, Armonk, NY, USA) statistical software. Descriptive data were reported in terms of mean, standard deviation (SD), and percentage. Skewness and kurtosis were calculated for the investigated variables, in order to verify their normal distribution. Differences were evaluated using the Student’s t test. Correlations were explored through the Pearson’s coefficient.

Univariate and multivariate linear regressions were performed to explore the association between the investigated variables. The PCS and the MCS were considered as the dependent variables, in both cross-sectional and longitudinal observations. The multivariate linear regressions included those variables that were significant at the univariate regressions. Furthermore, multivariate regression models were developed by hierarchically including the independent variables; we firstly included the sociodemographic variables (e.g., age, gender), then the clinical ones (e.g., cognitive status, frailty status) since they are established contributors to HRQoL; finally, we tested the potential contribution of the psychological variables to explain the model. The FI was considered as an index of subjects’ global health.

Values of *p* < 0.05 were considered statistically significant.

## 3. Results

### 3.1. Baseline Characteristics of the Sample

The original sample consisted of 141 elderly outpatients. The mean age of the sample was approximatively eighty years (80.31 ± 6.84); the outpatients were predominantly females, corresponding to 70% of the total sample; the majority of the outpatients were married (50.4%) or widow/er (39.7%).

The outpatients who were classified as frail, according to the calculated FI, exhibited significantly lower PCS (45.53 ± 6.30; *p* < 0.001) and MCS (45.77 ± 7.50; *p* < 0.001) scores compared to those who were classified as not frail (PCS = 54.13 ± 11.01; MCS = 53.91 ± 10.47).

Furthermore, frail outpatients showed significantly lower levels of dispositional optimism (LOT-R = 16.25 ± 5.3; *p* = 0.002), and FREE Suppression (3.60 ± 0.74; *p* = 0.034) compared to not frail outpatients (LOT-R = 20.04 ± 5.23; FREE Suppression = 4.01 ± 0.86).

The main clinical and sociodemographic baseline features of the sample are summarized in Table 1.

### 3.2. Cross-Sectional Results

We performed a preliminary correlation analysis using the data obtained at baseline (from October 2018 to October 2019). The PCS score was positively correlated to LOT-R (*r* = 0.390; *p* < 0.001), and to FREE Suppression (*r* = 0.313; *p* = 0.004). The MCS score was positively correlated to LOT-R (*r* = 0.525; *p* < 0.001), FREE Suppression (*r* = 0.266; *p* = 0.014), and to the FREE Total (*r* = 0.267; *p* = 0.014). The main correlations are extensively reported in Table 2.

We further analyzed the data obtained at baseline (from October 2018 to October 2019) by performing univariate and multivariate linear regressions, in order to find variables significantly associated with PCS and MCS, and to test the association of the psychological factors with HRQoL.

The multivariate linear regression model for PCS was hierarchically developed, and included gender and education (Step 1: R^2^ adjusted = 0.043; F = 2.892; *p* = 0.061), FI (Step 2: R^2^ adjusted = 0.210; F = 8.448; *p* < 0.001), and ultimately LOT-R and FREE Suppression (Step 3: R^2^ adjusted = 0.280; F = 7.529; *p* < 0.001), since these variables were significantly associated with PCS in the univariate regressions. The final step of the multivariate model showed that FREE Suppression (β = 0.203; *p* = 0.047) and FI (β = −0.323; *p* = 0.003) were significantly associated with PCS.

Similarly, the multivariate linear regression model for MCS was hierarchically developed, including those variables significantly associated with MCS in the univariate regressions. Accordingly, none of the sociodemographic factors were included in the multivariate model for MCS; MMSE was included in the first step (R^2^ adjusted = 0.016; F = 2.335; *p* = 0.13), FI was included in the second step (R^2^ adjusted = 0.128; F = 7.177; *p* = 0.001), and LOT-R and FREE Suppression were included in the third step (R^2^ adjusted = 0.308; F = 10.359; *p* < 0.001). The final step was significantly explained by LOT-R (β = 0.441; *p* < 0.001) and FI (β = −0.273; *p* = 0.023).

The univariate and multivariate linear regression models are available in detail as Supplementary Materials (Tables S2–S5).

### 3.3. Characteristics of the Sample Evaluated during the COVID-19 Outbreak

From the original sample, we were able to contact by telephone 115 outpatients; however, eleven declined to undergo the evaluation; we were not able to contact the remaining twenty-six outpatients, as they could not be reached by telephone.

The final number of outpatients included in the follow-up evaluation was 104 (mean age 80.26 ± 6.39); the majority of the patients were women (65.7%); the mean educational level was 7.12 years. Outpatients exhibited significantly worse PCS (46.42 ± 10.28; *p* < 0.001) and MCS (46.35 ± 10.06; *p* < 0.001) scores compared to their corresponding scores reported at baseline.

The principal characteristics of the sample evaluated in April 2020, as well as the differences between baseline and longitudinal HRQoL levels, are summarized in Table 3.

### 3.4. Univariate and Multivariate Linear Regressions for PCS and MCS Assessed at Follow-Up

We developed linear and multivariate regression models to test the association of baseline sociodemographic, clinical and psychological factors with the two HRQoL indexes assessed at follow-up, namely PCS and MCS. The univariate regression models at follow-up substantially confirmed the previous cross-sectional evidence.

Similar to the cross-sectional analyses, we developed a multivariate regression model for PCS, by hierarchically including gender and education (Step 1: R^2^ adjusted = 0.051; F = 3.270; *p* = 0.043), FI (Step 2: R^2^ adjusted = 0.282; F = 11.988; *p* < 0.001), LOT-R and FREE Suppression (Step 3: R^2^ adjusted = 0.358; F = 10.361; *p* < 0.001). In the final step of the model, FREE Suppression (β = 0.211; *p* = 0.029) and FI (β = −0.391; *p* < 0.001) were confirmed as two significant predictors of PCS.

The multivariate linear regression model for MCS was developed by hierarchically including MMSE (Step 1: R^2^ adjusted = 0.029; F = 3.55; *p* = 0.063), FI (Step 2: R^2^ adjusted = 0.191; F = 10.894; *p* < 0.001), LOT-R and FREE (Step 3: R^2^ adjusted = 0.399; F = 14.914; *p* < 0.001). Unlike the previous cross-sectional evidence, none of the two indexes of expressive flexibility was significantly associated with MCS at follow-up; nonetheless, the total FREE score was significantly associated with MCS at follow-up. The final step of the model confirmed LOT-R (β = 0.417; *p* = 0.001) and FI (β = −0.349; *p* = 0.002) as two predictors of MCS at follow-up. Additionally, the total FREE score was a new psychological predictor of MCS at follow-up (β = 0.215; *p* = 0.015).

The univariate linear regressions for both PCS and MCS are available as Supplementary Materials (Tables S6 and S7, respectively). The multivariate regression models for both longitudinal PCS and MCS are summarized in Table 4 and Table 5, respectively.

## 4. Discussion

The present study aimed to investigate the contribution of psychological factors in predicting HRQoL during the COVID-19 outbreak, in a sample of elderly outpatients. Dispositional optimism and expressive flexibility were found at baseline as variables cross-sectionally associated with the physical and the mental components of HRQoL; similarly, baseline dispositional optimism and baseline expressive flexibility were tested as potential predictors of the HRQoL assessed during the COVID-19 outbreak.

In line with the main findings of the study, baseline higher levels of dispositional optimism (measured through the LOT-R) were associated with better baseline MCS; additionally, baseline dispositional optimism was confirmed as a psychological predictor of the HRQoL mental component assessed at follow-up. Furthermore, subjects’ ability to flexibly suppress emotion expression (expressed by the FREE Suppression index) contributed to explaining the physical component of HRQoL, both at baseline and at follow-up. In addition, the FREE Total score, which reflects the comprehensive ability to flexibly regulate emotion expression, was found to predict the mental component of HRQoL at follow-up.

Optimism has been originally defined as one of the two sides of life orientation (the latter being pessimism), and it has been suggested as a contributor to individual’s psychological adaptability [17]. Our findings appear in line with previous studies, which have discussed the impact of dispositional optimism on physical health and individual wellbeing in adult populations [40,41], and its beneficial role for the treatment of subjects suffering from chronic medical conditions, such as cardiovascular diseases, cancer, diabetes, and neurological pathologies [42]. In this perspective, the role of dispositional optimism might be to motivate the individual to maintain pro-health behaviors, with positive consequences on physical health and HRQoL [43]. Consistent with this conceptualization, our findings further extend the beneficial role of dispositional optimism on the HRQoL of the elderly population, which has considerably suffered both the physical and the psychological consequences of the COVID-19 pandemic [44]. Compared to the univariate regression analyses, in which the LOT-R was associated with both PCS and MCS, the further multivariate model highlighted its significant impact on the elderly’s mental component of HRQoL, both at baseline and at follow-up. The items of the SF-12 that are related to the MCS record a possible decrease in performances at work and in carrying out daily activities, due to the subject’s emotional or mental state (e.g., depressed or anxious mood, decrease in concentration). Our findings could suggest that, while the physical component of the HRQoL in the elderly may be weakened by various age-related factors, an attitude oriented towards optimism could instead help the elderly to maintain a positive psychological and mental state even in particularly challenging situations, like the pandemic emergency.

Expressive flexibility is considered a further relevant factor able to promote resilience, as it contributes to maintaining a stable balance after exposure to stressful situations, and it favors a better individual adaptation [45]. According to this theoretical framework, adaptation depends on the individual ability to flexibly enhance or suppress emotion expression, in accordance with different contextual demands [24]. The positive role of this regulatory process has been previously revealed in healthy subjects, and in people suffering from posttraumatic disorders [46,47,48]. To the best of our knowledge, the investigation of expressive flexibility in the elderly population, and in association with their HRQoL, is a novel topic of research. The administration of the FREE scale to elderly subjects might be facilitated since it is a scenario-based scale, which does not require participants to possess an exact awareness of their own abilities [49]. In line with its known positive impact on mental health after adverse events, our findings additionally suggest that flexible emotion regulation might also promote a better perception of HRQoL in the elderly during the current COVID-19 pandemic. In our study, expressive flexibility had a different impact on PCS and MCS among the elderly. Specifically, we found that the FREE Suppression index was the only significant predictor of the HRQoL physical component. In addition, we showed that the FREE Total score significantly predicted the MCS. We interpreted the significant contribution of the FREE Total score on the mental component of elderly’s HRQoL in line with the adopted theoretical framework, according to which a flexible regulation of emotional expression has a positive effect on mental health, especially after adverse events [45]. The different contributions of suppression and enhancement abilities on HRQoL represents a challenging topic of debate. The association between FREE Suppression and PCS denotes an original starting point for future research, especially in the context of elderly patients. As we have highlighted in the premises of this study, most of the studies on the evaluation of expressive flexibility have involved non-clinical populations (e.g., healthy subjects, adolescents, veterans). Through our results, we can hypothesize and extend the contribution of suppression abilities to the physical domains of HRQoL, and not only to psychological health. However, these interpretations are strictly circumscribed by the nature of our sample, i.e., elderly outpatients; therefore, we hope that future studies will better clarify the contribution of expressive flexibility on the physical and mental health of a population as complex as the elderly.

The findings of our study also highlighted the joint contribution of both psychological and functional factors on elderly’s HRQoL, as suggested by the evaluation of frailty status. Frailty is generally defined as a clinical condition that exposes individual to an increased vulnerability to developing negative health-related outcomes, such as disability, hospitalization, and death, due to the occurrence of stressor events. Indeed, the accurate evaluation of frailty in the elderly population still represents a relevant public health need [50].

The interaction between HRQoL and frailty has generated a growing scientific interest, especially in the context of elderly subjects. Consistently, recent studies have showed a significant inverse association between HRQoL and frailty, as subjects showing a higher degree of frailty exhibited worse levels of physical and mental HRQoL [51,52]. Since we included outpatients from the Geriatric Clinic, we needed to capture the complexity of their health status, rather than solely their physical frailty; therefore, we evaluated frailty by calculating a FI, which is an expression of global health status, since it is based on the contribution of variables of different nature (see Table S1). We did not aim to investigate psychological or a social frailty, as defined for instance by the Gobbens model of frailty [53]. We needed a concept of frailty wider than merely physical, but still referring to age-related health. Therefore, we were able to develop our regression model in which frailty could be investigated together with psychological factors, whose joint contribution on HRQoL we wanted to explore.

The progressive restrictions due to the COVID-19 outbreak have dramatically unsettled our daily lives, forcing us to maintain social distance from friends and relatives, which inevitably leads to a psychological burden that is often not easy to sustain [54,55]. In this context, the elderly represented one of the populations most exposed to the direct and indirect consequences of the COVID-19 outbreak.

Aging is characterized as a complex process, resulting from several interactions between biological, psychological, and social factors, which can contribute in different ways to trace different trajectories of aging. Indeed, each of these components may concur as a protective factor towards physiological, or even to a successful, aging, as well as a vulnerability factor that progressively exposes the individual to reduced independence [56] and increased risk of adverse age-related outcomes [57,58,59,60]. Therefore, the implementation of preventive strategies should be aimed at facing the consequences of the pandemic not only on the physical health of the elderly [61] but also on their HRQoL and their psychological wellbeing.

Psychological resilience consists of the positive strength to overcome and adapt to difficulties or stressful situations; additionally, it is proved to be a relevant factor able to contribute to reduce emotional distress and to improve psychological wellbeing during the COVID-19 outbreak [30,31]. Previous studies on elderly subjects have highlighted that high levels of psychological resilience predict better psychological wellbeing, improve social relationships and favor health-oriented behaviors [15]. An accurate investigation of psychological factors able to promote resilience might be particularly important among the elderly, due to the presence of psychological, social, physical, and socioeconomic stressors they have to face. With this perspective, dispositional optimism and expressive flexibility could be considered as novel targets of interest, especially in the elderly population.

The investigation of psychological aspects could be justified within the context of a necessary multidimensional approach in elderly subjects, in which both physical [62] and psychological interventions [14] might have a positive impact on perceived HRQoL, especially during the COVID-19 pandemic. Furthermore, interventions aimed at improving or strengthening psychological factors, such as optimism or expressive flexibility, could be further facilitated by the implementation of telematics protocols, in order to overcome any difficulty in carrying them out in the presence of the participants [63].

The study presents some limitations that should be addressed by future research. The first is the relatively small sample size, which also did not allow us to investigate in detail the presence of gender differences. Moreover, the clinical setting of the study was an outpatients Clinic, which might not be fully representative of the studied population. Further studies with larger samples are encouraged in order to better confirm these preliminary findings.

Despite these limitations, in line with current scientific interests, the study focused on topics of considerable relevance, such as the psychological evaluation of elderly subjects during the COVID-19 outbreak, and the specific concern about its contribution to their HRQoL. Precisely, the evaluation of both dispositional optimism and expressive flexibility is original in the context of elderly outpatients and in association with their HRQoL. The investigation of two psychological constructs with robust and established theoretical backgrounds can be considered a further strength.

## 5. Conclusions

The negative impact of the COVID-19 outbreak on HRQoL in the elderly is well documented. Nonetheless, the contribution of psychological factors to HRQoL in the elderly is an actual and open topic of debate. The results of our study showed that dispositional optimism and expressive flexibility significantly contributed to the level of perceived HRQoL in a sample of elderly outpatients. The positive role of dispositional optimism has been found in several chronic medical conditions; our findings particularly discussed its contribution to predict the mental component of HRQoL in elderly subjects during the current pandemic. Expressive flexibility is able to reduce psychological distress in the community population; however, to the best of our knowledge, the investigation of expressive flexibility in an elderly population, and in association with their HRQoL, is an original topic of research. In this context, our study suggested the predictive role of suppression abilities, and in general of expressive flexibility, on HRQoL in elderly subjects.

The multidimensional nature of HRQoL acquires an important meaning in the context of elderly subjects, due to the contribution of different factors (e.g., physical, cognitive, social, and psychological). From a psychogeriatric perspective, the accurate assessment of psychological factors, such as dispositional optimism and expressive flexibility, might help physicians and psychologists to recognize additional patients’ vulnerabilities. The strengthening of dispositional optimism and expressive flexibility might additionally represent a novel target for psychological interventions, with the aim of helping the elderly to positively react in the presence of a dramatic event such as a pandemic, and partially contributing to the improvement of their HRQoL.

## Figures and Tables

**Table 1:** Main sociodemographic and clinical baseline characteristics of the sample.

**Sample (N = 141)**						
**Prevalences**						
			N		%	
Gender male			42		29.8	
Gender female			99		70.2	
Married			71		50.4	
Widow/er			56		39.7	
Other marital status			14		9.9	
Frail			71		50.4	
Not frail			70		49.6	
**Descriptives**						
	**Mean**	**SD**	**Skewness**	**SE**	**Kurtosis**	**SE**
Age	80.31	6.84	−0.309	0.204	0.366	0.406
Education	7.09	3.83	1.105	0.204	0.618	0.406
Mini Mental State Examination (MMSE)	22.60	4.52	−0.547	0.204	−0.340	0.406
Frailty Index (FI)	0.25	0.11	0.195	0.204	−0.914	0.406
Life Orientation Test (LOT-R)	18.20	5.58	−0.245	0.243	−0.470	0.481
Flexible Regulation of Emotional Expression (FREE) Suppression	3.84	0.83	0.161	0.261	−0.80	0.517
FREE Enhancement	3.91	0.92	−0.84	0.261	−0.25	0.517
FREE Total	7.06	1.55	−0.203	0.261	−0.85	0.517
Physical Component Summary (PCS)	50.00	10.00	0.810	0.237	0.210	0.469
Mental Component Summary (MCS)	49.99	9.99	0.582	0.237	0.310	0.469

SD = Standard deviation; SE = Standard Error.

**Table 2:** Main correlations between the variables investigated at baseline.

	PCS		MCS	
	*r*	*p*	*r*	*p*
Age	0.04	0.68	0.083	0.4
Gender	−0.260	**0.008**	−0.149	0.13
Education	0.195	**0.047**	0.062	0.53
LOT-R	0.390	**<0.001**	0.525	**<0.001**
FREE Suppression	0.313	**0.004**	0.266	**0.014**
FREE Enhancement	0.083	0.448	0.183	0.09
FREE	0.164	0.13	0.267	**0.014**
FI	−0.455	**<0.001**	−0.389	**<0.001**
MMSE	0.135	0.17	0.201	**0.04**

Significant *p* values are reported in bold.

**Table 3:** Principal sociodemographic and clinical characteristics of the sample evaluated during the COVID-19 outbreak in April.

**Sample (N = 104)**				
Age (years; mean ± SD)			80.26 (±6.39)	
Gender (n; %)			Male: 33 (34.3)	Female: 71 (65.7)
Education (years; mean ± SD)			7.12 (±3.87)	
FI (mean ± SD)			0.24 (0.10)	
Frailty status Frail (n; %) Not frail (n; %)			50 (48.1)	
			54 (51.9)	
	**Baseline (t0)**	**Longitudinal (t1)**	**z (t1−t0)**	***p***
PCS	50 (±10)	46.42 (±10.28)	−8.67	<0.001
MCS	49.99 (±9.99)	46.35 (±10.06)	−8.27	<0.001

SD: Standard deviation.

**Table 4:** Multivariate linear regression for PCS assessed at follow-up.

	**R^2^**	**Adjusted R^2^**	**F**	***p***
**Step 1**	0.074	0.051	3.270	**0.043**
	**SE (B)**	**β**	***t***	***p***
Gender	2.494	−0.121	−1.079	0.28
Education	0.301	0.208	1.860	0.06
	**R^2^**	**Adjusted R^2^**	**F**	***p***
**Step 2**	0.307	0.282	11.988	**<0.001**
	**SE (B)**	**β**	***t***	***p***
Gender	2.174	−0.155	−1.584	0.11
Education	0.269	0.083	0.830	0.40
FI	9.997	−0.498	−5.227	**<0.001**
	**R^2^**	**Adjusted R^2^**	**F**	***p***
**Step 3**	0.396	0.358	10.361	**<0.001**
	**SE (B)**	**β**	***t***	***p***
Gender	2.113	−0.152	−1.60	0.11
Education	0.267	0.049	0.489	0.62
FI	10.284	−0.391	−3.995	**<0.001**
LOT-R	0.181	0.195	1.978	0.051
FREE Suppression	1.192	0.211	2.22	**0.029**

SE (B): Standard error (B); significant *p* values are reported in bold.

**Table 5:** Multivariate linear regression for MCS assessed at follow-up.

	**R^2^**	**Adjusted R^2^**	**F**	***p***
**Step 1**	0.041	0.029	3.55	0.063
	**SE (B)**	**β**	***t***	***p***
MMSE	0.283	0.203	1.884	0.063
	**R^2^**	**Adjusted R^2^**	**F**	***p***
**Step 2**	0.210	0.191	10.894	**<0.001**
	**SE (B)**	**β**	***t***	***p***
MMSE	0.319	−0.094	−0.777	0.43
FI	12.155	−0.507	−4.187	**<0.001**
	**R^2^**	**Adjusted R^2^**	**F**	***p***
**Step 3**	0.427	0.399	14.914	**<0.001**
	**SE (B)**	**β**	***t***	***p***
MMSE	0.278	−0.145	−1.373	0.17
FI	10.869	−0.349	−3.223	**0.002**
LOT-R	0.164	0.417	4.462	**0.001**
FREE	0.560	0.215	2.486	**0.015**

SE (B): Standard error (B); significant *p* values are reported in bold.

## Data Availability

The raw data supporting the conclusions of this article will be made available on request by the corresponding author, without undue reservation.

## Supplementary Materials

The following are available online at https://www.mdpi.com/1660-4601/18/4/1698/s1. Table S1: Variables considered in the calculated Frailty Index (FI); Table S2: Univariate linear regressions for baseline PCS; Table S3: Univariate linear regressions for baseline MCS; Table S4: Multivariate linear regression for baseline PCS; Table S5: Multivariate linear regression for baseline MCS; Table S6: Univariate linear regressions for longitudinal PCS; Table S7: Univariate linear regressions for longitudinal MCS.
