# Supplementary material for: Expressive Flexibility and Dispositional Optimism Contribute to the Elderly’s Resilience and Health-Related Quality of Life during the COVID-19 Pandemic
Source: Int J Environ Res Public Health. 2021 Feb 10;18(4):1698. doi: 10.3390/ijerph18041698 (PMC7916547; doi:10.3390/ijerph18041698)
Supplement: Supplementary file 1 [file ijerph-18-01698-s001.zip › Table S1.pdf]

Table S1. Variables considered in the calculated Frailty Index (FI).

Hospitalization	Pain	Urinary incontinence	Heart failure	Cerebrovascular disease
Fractures	Bathing	Faecal incontinence	Chronic Obstructive Pulmonary Disease	Handgrip strength
Caregiver	Dressing	Telephone	BMI	Parkinsonism
Cognitive status	Walking	Drugs	Cancer	Gait speed
Malnutrition	Getting up / Sitting down	Hypertension	Cirrhosis	Medications
Dehydration	Feeding	Diabetes	Chronic kidney failure	Benzodiazepines
Oral health	Toileting	Heart disease	Obesity	Neuroleptics
			Total number of detected deficits	*Frailty Index*
			__ / 35	

Note: The FI is expressed as a ratio of health deficits present to the total number of deficits considered; the greater the number of health deficits, the higher the degree of frailty. According to this approach, patients with a FI ≥0.25 are commonly considered frail.
