# Supplementary material for: Expressive Flexibility and Dispositional Optimism Contribute to the Elderly’s Resilience and Health-Related Quality of Life during the COVID-19 Pandemic
Source: Int J Environ Res Public Health. 2021 Feb 10;18(4):1698. doi: 10.3390/ijerph18041698 (PMC7916547; doi:10.3390/ijerph18041698)
Supplement: Supplementary file 1 [file ijerph-18-01698-s001.zip › Table S2.pdf]

Table S2. Univariate linear regression for baseline PCS

	B	SE(B)	β	*p*	95% CI	
					Lower	Upper
Age	0.062	0.155	0.040	0.68	-0.245	0.369
Gender	-5.565	2.044	-0.260	***0.008***	-9.620	-1.510
Education	0.504	0.251	0.195	***0.047***	0.007	1.002
FI	-44.523	8.617	-0.455	***<0.001***	-61.61	-27.43
LOT-R	0.705	0.169	0.390	***<0.001***	0.370	1.041
FREE	1.095	0.723	0.164	0.134	-0.334	2.534
FREE_enha	0.941	1.233	0.083	0.448	-1.512	3.394
FREE_supp	3.896	1.296	0.313	***0.004***	1.318	6.475

Abbreviations: LOT-R: Life Orientation Test-Revised; FREE: Flexible Regulation of Emotional Expression; FREE_supp: Suppression; FREE_enha: Enhancement; PCS: Physical Component Summary; FI: Frailty Index.
