# Supplementary material for: Expressive Flexibility and Dispositional Optimism Contribute to the Elderly’s Resilience and Health-Related Quality of Life during the COVID-19 Pandemic
Source: Int J Environ Res Public Health. 2021 Feb 10;18(4):1698. doi: 10.3390/ijerph18041698 (PMC7916547; doi:10.3390/ijerph18041698)
Supplement: Supplementary file 1 [file ijerph-18-01698-s001.zip › Table S3.pdf]

Table S3. Univariate linear regressions for MCS.

	**B**	**SE**	**β**	***p***	**95% CI**	
					Lower	Upper
Age	0.130	0.154	0.083	0.40	-0.176	0.437
Gender	-3.179	2.094	-0.149	0.13	-7.331	0.974
Education	0.160	0.255	0.062	0.53	-0.346	0.66
MMSE	0.464	0.223	0.201	***0.04***	0.021	0.907
LOT-R	0.942	0.155	0.525	***<0.001***	0.634	1.249
FREE	1.748	0.692	0.267	***0.014***	0.370	3.125
FREE_enha	2.025	1.192	0.183	0.093	-0.347	4.396
FREE_supp	3.245	1.289	0.266	***0.014***	0.681	5.810
FI	-38.00	8.917	-0.389	***<0.001***	-55.68	-20.31

Abbreviations: LOT-R: Life Orientation Test-Revised; FREE: Flexible Regulation of Emotional Expression; FREE_supp: Suppression; FREE_enha: Enhancement; MCS: Mental Component Summary; FI: Frailty Index; MMSE: Mini Mental State Examination.
