# Supplementary material for: Expressive Flexibility and Dispositional Optimism Contribute to the Elderly’s Resilience and Health-Related Quality of Life during the COVID-19 Pandemic
Source: Int J Environ Res Public Health. 2021 Feb 10;18(4):1698. doi: 10.3390/ijerph18041698 (PMC7916547; doi:10.3390/ijerph18041698)
Supplement: Supplementary file 1 [file ijerph-18-01698-s001.zip › Table S4.pdf]

Table S4. Multivariate linear regression for PCS

	**R^2**	**Adjusted R^2**	**F**	***p***
**Step 1**	0.066	0.043	2.892	0.061
	**SE(B)**	**β**	**T**	***p***
Gender	2.478	-0.147	-1.303	0.19
Education	0.299	0.169	1.507	0.13
	**R^2**	**Adjusted R^2**	**F**	***p***
**Step 2**	0.238	0.210	8.448	***<0.001***
	**SE(B)**	**β**	**T**	***p***
Gender	2.256	-0.176	-1.716	0.09
Education	0.280	0.062	0.589	0.55
FI	10.37	-0.428	-4.282	***<0.001***
	**R^2**	**Adjusted R^2**	**F**	***p***
**Step 3**	0.323	0.280	7.529	***<0.001***
	**SE(B)**	**β**	**T**	***p***
Gender	2.213	-0.172	-1.713	0.09
Education	0.280	0.029	0.278	0.78
FI	10.77	-0.323	-3.113	***0.003***
LOT-R	0.190	0.193	1.850	0.06
FREE_supp	1.249	0.203	2.023	***0.047***

Abbreviations: PCS: Physical Component Summary; LOT-R: Life Orientation Test-Revised; FREE: Flexible Regulation of Emotional Expression; FREE_supp: Suppression; FI: Frailty Index.
