# Supplementary material for: Expressive Flexibility and Dispositional Optimism Contribute to the Elderly’s Resilience and Health-Related Quality of Life during the COVID-19 Pandemic
Source: Int J Environ Res Public Health. 2021 Feb 10;18(4):1698. doi: 10.3390/ijerph18041698 (PMC7916547; doi:10.3390/ijerph18041698)
Supplement: Supplementary file 1 [file ijerph-18-01698-s001.zip › Table S5.pdf]

Table S5. Multivariate linear regression for MCS

	R^2	Adjusted R^2	F	*p*
**Step 1**	0.027	0.016	2.335	0.13
	**SE(B)**	**β**	**t**	***p***
MMSE	0.289	0.165	1.528	0.13
	R^2	Adjusted R^2	F	*p*
**Step 2**	0.149	0.128	7.177	***0.001***
	**SE(B)**	**β**	**t**	***p***
MMSE	0.336	-0.086	-0.687	0.49
FI	12.790	-0.430	-3.423	***0.001***
	R^2	Adjusted R^2	F	*p*
**Step 3**	0.341	0.308	10.359	***<0.001***
	**SE(B)**	**β**	**t**	***p***
MMSE	0.306	-0.137	-1.198	0.23
FI	11.953	-0.273	-2.324	***0.023***
LOT-R	0.180	0.441	4.360	***<0.001***
FREE_supp	1.165	0.105	1.099	0.27

Abbreviations: MMSE: Mini Mental State Examination; LOT-R: Life Orientation Test-Revised; FREE: Flexible Regulation of Emotional Expression; FREE_supp: Suppression; MCS: Mental Component Summary; FI: Frailty Index.
