# Supplementary material for: Expressive Flexibility and Dispositional Optimism Contribute to the Elderly’s Resilience and Health-Related Quality of Life during the COVID-19 Pandemic
Source: Int J Environ Res Public Health. 2021 Feb 10;18(4):1698. doi: 10.3390/ijerph18041698 (PMC7916547; doi:10.3390/ijerph18041698)
Supplement: Supplementary file 1 [file ijerph-18-01698-s001.zip › Table S6.pdf]

Table S6: Univariate linear regressions for longitudinal PCS.

	B	SE(B)	β	*p*	95% CI	
					Lower	Upper
Age	-0.035	0.159	-0.022	0.82	-0.35	0.281
Gender	-5.484	2.108	-0.249	***0.011***	-9.66	-1.30
Education	0.646	0.255	0.243	***0.013***	0.141	1.152
LOT-R	0.759	0.172	0.410	***<0.001***	0.418	1.099
FREE	1.322	0.727	0.196	0.07	-0.123	2.76
FREE_enha	1.160	1.244	0.102	0.35	-1.315	3.635
FREE_supp	4.279	1.297	0.340	***0.001***	1.689	6.859
FI	-55.47	8.29	-0.552	***<0.001***	-71.93	-39.01

Abbreviations: PCS: Physical Component Summary; LOT-R: Life Orientation Test-Revised; FREE: Flexible Regulation of Emotional Expression; FREE_enha: Enhancement; FREE_supp: Suppression; FI: Frailty Index.
