# Supplementary material for: Expressive Flexibility and Dispositional Optimism Contribute to the Elderly’s Resilience and Health-Related Quality of Life during the COVID-19 Pandemic
Source: Int J Environ Res Public Health. 2021 Feb 10;18(4):1698. doi: 10.3390/ijerph18041698 (PMC7916547; doi:10.3390/ijerph18041698)
Supplement: Supplementary file 1 [file ijerph-18-01698-s001.zip › Table S7.pdf]

Table S7: Univariate linear regressions for longitudinal MCS

	**B**	**SE(B)**	**β**	***p***	**95% CI**	
					Lower	Upper
Age	0.032	0.156	0.020	0.83	-0.277	0.341
Gender	-3.115	2.108	-0.145	0.14	-7.296	1.065
Education	0.342	0.255	0.132	0.18	-0.164	0.847
LOT-R	0.961	0.155	0.532	***<0.001***	0.653	1.269
FREE	2.181	0.667	0.338	***0.002***	0.854	3.507
FREE_enha	2.543	1.163	0.233	***0.03***	0.229	4.856
FREE_supp	4.155	1.238	0.346	***0.001***	1.693	6.618
MMSE	0.675	0.219	0.291	***0.003***	0.240	1.110
FI	-47.27	8.53	-0.481	***<0.001***	-64.21	-30.33

Abbreviations: MCS: Mental Component Summary; LOT-R: Life Orientation Test-Revised; FREE: Flexible Regulation of Emotional Expression; FREE_enha: Enhancement; FREE_supp: Suppression; FI: Frailty Index; MMSE: Mini Mental State Examination.
